# Impact of superimposed nephrological care to guidelines-directed management by primary care physicians of patients with stable chronic kidney disease: a randomized controlled trial

**DOI:** 10.1186/s12882-020-01747-3

**Published:** 2020-04-09

**Authors:** Patrick Saudan, Belen Ponte, Nicola Marangon, Chantal Martinez, Lena Berchtold, David Jaques, Thomas Ernandez, Sophie de Seigneux, Sebastian Carballo, Thomas Perneger, Pierre-Yves Martin

**Affiliations:** 1 Nephrology Unit, Department of Medicine, Geneva University Hospitals, Geneva, Switzerland; 2 Service of General Internal Medicine, Department of Medicine, Geneva University Hospitals, Geneva, Switzerland; 3 Division of Clinical Epidemiology, Geneva University Hospitals, Geneva, Switzerland

**Keywords:** Co-management, Primary care, Chronic kidney disease, Quality of life, Prognosis

## Abstract

**Background:**

Optimal clinical care of patients with chronic kidney disease (CKD) requires collaboration between primary care physicians (PCPs) and nephrologists. We undertook a randomised trial to determine the impact of superimposed nephrologist care compared to guidelines-directed management by PCPs in CKD patients after hospital discharge.

**Methods:**

Stage 3b-4 CKD patients were enrolled during a hospitalization and randomised in two arms: Co-management by PCPs and nephrologists (interventional arm) versus management by PCPs with written instructions and consultations by nephrologists on demand (standard care). Our primary outcome was death or rehospitalisation within the 2 years post-randomisation. Secondary outcomes were: urgent renal replacement therapy (RRT), decline of renal function and decrease of quality of life at 2 years.

**Results:**

From November 2009 to the end of June 2013, we randomised 242 patients. Mean follow-up was 51 + 20 months. Survival without rehospitalisation, GFR decline and elective dialysis initiation did not differ between the two arms. Quality of life was also similar in both groups*.* Compared to randomised patients, those who either declined to participate in the study or were previously known by nephrologists had a worse survival.

**Conclusion:**

These results do not demonstrate a benefit of a regular renal care compared to guided PCPs care in terms of survival or dialysis initiation in CKD patients. Increased awareness of renal disease management among PCPs may be as effective as a co-management by PCPs and nephrologists in order to improve the prognosis of moderate-to-severe CKD.

**Trial registration:**

This study was registered on June 29, 2009 in clinicaltrials.gov (NCT00929760) and adheres to CONSORT 2010 guidelines.

## Background

The prevalence of chronic kidney disease (CKD), and subsequent end stage renal disease (ESRD) is on the rise, particularly in patients older than 65 years [1]. Despite constant improvements of renal replacement therapies (RRT), the morbidity and mortality of these patients remain high, with a survival of ESRD patients as low as 60 and 30% at 1 and 5 years, respectively [2, 3].

Many factors have been related to this poor outcome including late referral to nephrologists [2, 4–6]. The two most reported explanations for the association of a late referral with a poor prognosis are a belated intervention by specialists and complications related to emergency dialysis. A delay in specialised care exposes the patients to possible under-treatment of CKD complications such as cardiovascular disease (CVD), anaemia, bone and mineral disorders and increased susceptibility to infections, or to emergency implementation of RRT, which is associated with increased morbidity and mortality, and duration of hospitalisation [7]. In addition, initiation of urgent haemodialysis (HD) instead of planned HD deprives the patient of the possibility to choose another type of RRT such as peritoneal dialysis (PD) [4, 8–11] or pre-emptive renal transplantation (TX) [12]. The European Best Practice Guidelines (EBPG) recommend that patients should be referred to nephrologists when creatinine clearance (CCl) < 60 ml/min and imperatively when CCl < 30 ml/min [13]. However, “real life” practice shows that early referral to nephrologists of CKD patients is uncommon [3–6]. Impact of guidelines on PCPs is difficult to evaluate but is probably limited as most recommendations appear in specialised journals [14]. Other important barriers to timely referral are: patient’s unawareness, fear of dialysis and PCPs’ underestimation of CKD severity. There is also no financial incentive for early referral, which hinders the implementation of the guidelines among PCPs [14–16]. The practical implementation of early referral also remains questionable as it is unlikely that nephrologists would be able to provide regular follow up of all patients with CKD [15, 17, 18].

In addition, there is some doubt on the impact in reducing mortality in patients or decreasing ESRD occurrence in patients with CCl between 45 and 60 ml/min but without albuminuria [18, 19]. An observational study from East Kent in UK, found a prevalence of patients with moderate to severe CKD as high as 0.55%, using cut-off values: serum creatinine > 180 μmol/l in males and > 135 μmol/l in females [17]. In this study, 84.8% of the patients were unknown to local nephrology units and only 8.1% were referred to them during the 31.3 months follow-up. Median survivals of referred and unreferred patients were 29.1 and 27.4 months, respectively (*p* < 0.001). Interestingly, ESRD by itself accounted for only 4.8% of deaths whereas the majority of patients died from CVD, infections and cancer. Male sex, low glomerular filtration rate (GFR) and non-referral were associated with poor prognosis. The authors concluded that the referral of all identified patients would have led to a non-sustainable overload of nephrology care resources.

There is therefore a need for the development and implementation of new strategies through collaboration between PCPs and nephrologists. However, prior to the increase of medical resources devoted to renal care, assessment of the impact of specialised care in renal patients should be thoroughly evaluated.

We therefore aimed to determine the impact of specialised care by nephrologists (co-management) compared to guidelines-directed management by PCPs on survival, RRT planning and quality of life in CKD patients who were discharged from hospitalisation.

## Methods

### Study design

This was a single center prospective randomised study.

From November 2009 to the end of June 2013, patients with CKD were identified during their hospitalization at the Geneva University Hospitals, which is a tertiary center. A research nurse (CM) weekly screened all electronic patient files within hospital medical and surgical wards and potentially eligible patients were identified by their laboratory data. Those with CKD stage 3b-4 (eGFR between 44 and 15 ml/min/1.73m^2^) according to abbreviated MDRD-GFR formula and aged 18–80 years old, were eligible for the study.

Exclusion criteria included patients previously known by nephrologists or patients diagnosed with acute kidney injury (AKI) or those whose estimated life expectancy was< 1 year and those who refused or were unable to sign a written consent.

The patients who did not agree to participate and those already followed by a nephrologist were subsequently asked to sign a consent form allowing us to obtain annually post-discharge medical information (death, hospitalisation, initiation of urgent RRT, decline of renal residual function during the following 24 months after signature of consent form) from their PCPs/nephrologists.

### Randomisation

Those who agreed to participate were randomised in two arms. The randomization list was generated by computer, in blocks of random size. The 2 treatment arms were:

-Combined management PCPs- nephrologists with at least 4 nephrology visits/year.

(Nephrology Unit outpatient clinics). Our model of CKD care included a 30 min duration consultation with a nephrologist, during which a clinical examination an assessment of laboratory values were performed, with time also allocated to provide lifestyle and dietitian counselling. There was also a patient’s therapeutic education program led by nurses devoted to all patients (both arms of the study could benefit from it). This specific intervention was not evaluated in this study.

-Management by PCPs only*,* with written instructions from our nephrology unit based on KDOQI clinical practice guidelines [20] (Table 1). Treatment advice to PCPs was provided by our nephrology division on request, by email or by telephone. Patients with no PCP were followed after discharge at the outpatient clinics of Department of Community Medicine.

**Table 1:** Guidelines for PCPs on management of progression and complications of CKD, adapted from KDOQI clinical practice guidelines

**Blood pressure and proteinuria**	
First-line drug: ACEIs or ARBs in patients with proteinuria or diabetic nephropathy.	
Aim: BP ≤130/80 mmHg or ≤ 125/75 mmHg if proteinuria > 1 g/24 h; Proteinuria < 500 mg/24 h.	
**Diabetes**	
Use of antidiabetic drugs appropriate to renal function.	
Aim: Glycated Hb < 7%.	
**Dyslipidemia**	
Statins prescription.	
Aim: LDL cholesterol < 2.6 mmol/l.	
**Anemia**	
Iron supplementation; Erythropoietin prescription when appropriate.	
Aim: Hb 100–110 g/l.	
**Metabolic bone disease**	
Low-phosphate diet, prescription of phosphate binders, and vitamin D analogs when appropriate.	
Aim: Phosphate < 1.8 mmol/l, Calcium 2.2–2.6 mmol/l, PTH 14–21 pmol/l.	
**Metabolic acidosis**	
Prescribe oral sodium bicarbonate if serum bicarbonate < 22 mmol/l.	
Aim: Serum bicarbonate > 23 mmol/l.	
**Lifestyle changes**	
Medical and dietitian counseling, prescription of low-sodium and low-phosphate diets, low-protein, low potassium diet when appropriate.	
Aim: smoking cessation, increase of physical activity, and adapted diet to CKD stages.	

Neither PCPs or nephrologist were blinded to patient allocation or outcome.

Our research nurse collected the data in both groups. She had frequent contact with PCPs by mail or by phone calls to retrieve the requested information for the study.

### Outcomes

The primary (composite) outcome was death or emergency hospitalisation during the 24 months after inclusion.

Secondary outcomes were initiation of urgent RRT, decline of renal function and decrease of quality of life at 2 years after inclusion.

### Variables collected

Clinical, demographic and laboratory data (Table 2) were collected at inclusion time and at year 1 and 2 in patients who accepted the study. Modified Charlson score was used as comorbidity score [21]. Renal function decline was calculated by the difference between baseline and year 1 and 2 values in estimated glomerular filtration rate (eGFR) and measured creatinine clearances by 24-h urine collections.

**Table 2:** Time course of clinical, paraclinical and laboratory evaluations

Months	0	6	12	18	24
**Clinical evaluation**					
Charlson’s comorbidity score	X				
Clinical events (hospitalization, death, emergency RRT)			X		X
Kidney Disease Quality of Life questionnaire	X		X		X
**Paraclinical evaluation**					
Echocardiography (LVH and EF)	X		X		X
Targeted BP (24 h monitoring)	X		X		X
**Laboratory evaluation**					
24 h proteinuria and creatininuria	X		X		X
eGFR	X		X		X
Ferritin and transferrin saturation	X		X		X
B12 and folate	X		X		X
Albumin	X		X		X
PTH, calcium, phosphate, bicarbonate	X		X		X
BNP and NT pro-BNP	X		X		X
Cholesterol and triglycerides	X		X		X
CRP and IL-6	X		X		X

Rehospitalisation, RRT initiation and death were recorded with the help of the hospital and Civil registry databases.

Quality of life was assessed every year with the help of the French version of the Kidney Disease Quality of Life (KDQOL) questionnaire, which was recently validated in a French-speaking population [22]. The patients completed 38 items of the 11 kidney-specific scales of the KDQOL questionnaire, version 1.2 [23]. They did not complete the generic SF36 questionnaire. We added a numerical scale for health, rated between 0 (death or worst imaginable health) and 10 (perfect health), that was also included in the French translation [22]. KDQOL scores, computed according to the original publication were scaled between 0 (worst) and 100 (best) [23]. Because only a minority of patients received renal replacement therapy during the trial, we do not show results for the scales “Dialysis staff encouragement” and “Patient satisfaction”.

### Statistical analysis

Data analyses: values were expressed as means and standard deviation or median and interquartile ranges according to their distribution. Parametric and non-parametric tests were used to check for major dissimilarities between demographic and baseline characteristics of study groups. Significant *p* value was set at both-sides *p* < 0.05.  To compare the characteristics evolution over the follow-up between PCP and combined management, we first create a variable “post” to consider the time after randomization: post = 0 represents time before randomization, post = 1 is time after randomization at 1 year and 2 year. Then we used mixed linear and logistic regression to account for repetitive measures; participant’s characteristics were the dependent variables, time (“post”) and random group were the independent variables. We added an interaction term (post*random group) as an independent variable to measure whether there was a difference in the characteristics over time post-randomisation. We report the *p*-value of the interaction term in order to show differences between PCP and combined management. A significant interaction (p-value< 0.05) confirmed a difference in the evolution between the 2 groups after randomisation.

Kaplan-Meier survival curves were drawn for the univariate analysis of the main composite outcome (death or rehospitalisation) within the two years after randomisation. The log rank test was used to compare survival curves. A Cox proportional hazard model was used to investigate independent risk factors for the main composite outcome and implementation of emergency RRT. Age, gender, comorbidity score, eGFR were used as covariates. Randomised patients were also compared to the 2 observational cohorts (patients who declined and those already known by nephrologists) for survival by Kaplan-Meier analysis. Analyses were done based on intention to treat. The statistical analyses were performed with the use of SPSS version 25 (IBM, Armonk, NY) and STATA version 15 (Statacorp LP, College station, TX). The analysed datasets (randomised patients and those within the observational cohorts) are listed in the supplement files 1 and 2.

KDQOL analyses: We present means and standard deviations (SD) for the 9 other scales and for the self-rated health item, also scaled between 0 and 100 to facilitate comparisons, at 3 points in time, for the 2 trial arms. To capture the effect of treatment by the nephrologist (vs primary care physician) we used mixed linear models among patients who had a baseline assessment and at least one follow-up assessment. Because we saw no meaningful changes between the 2 follow-up assessments in exploratory analysis, we pooled the follow-up assessments and only sought to quantify a change from baseline. The models included a random intercept (*a*_0_) for each patient, to capture a patient’s tendency to return high or low scores. The form of the models was as follows:

$$ \mathrm{Score}={a}_0+{b}_1\ast \mathrm{group}+{b}_2\ast \mathrm{follow}-\mathrm{up}+{b}_3\ast \mathrm{group}\ast \mathrm{follow}-\mathrm{up} $$

The variable “group” was 0 for primary care physicians and 1 for co-management, and the variable “follow-up” was 0 at baseline and 1 at follow-up. The coefficient *b*_1_ represents the difference between the groups at baseline, *b*_2_ represents change at follow-up in the primary care physician group, and *b*_3_ represents the additional effect at follow-up of co-management. We only report *b*_3_ coefficients, which capture the effect tested in the trial.

### Sample size

We postulated that specialised renal care would decrease the primary endpoint prevalence from 30% (PCP group only) to 15% (PCP-nephrologist) within the study period. We calculated a sample size of 133 patients per arm (β = 0. 8, α < 0.05). Taking into account a 20% rate of refusal and loss of follow-up, we planned to enrol 320 patients during a 30-month period.

## Results

**Fig. 1:**
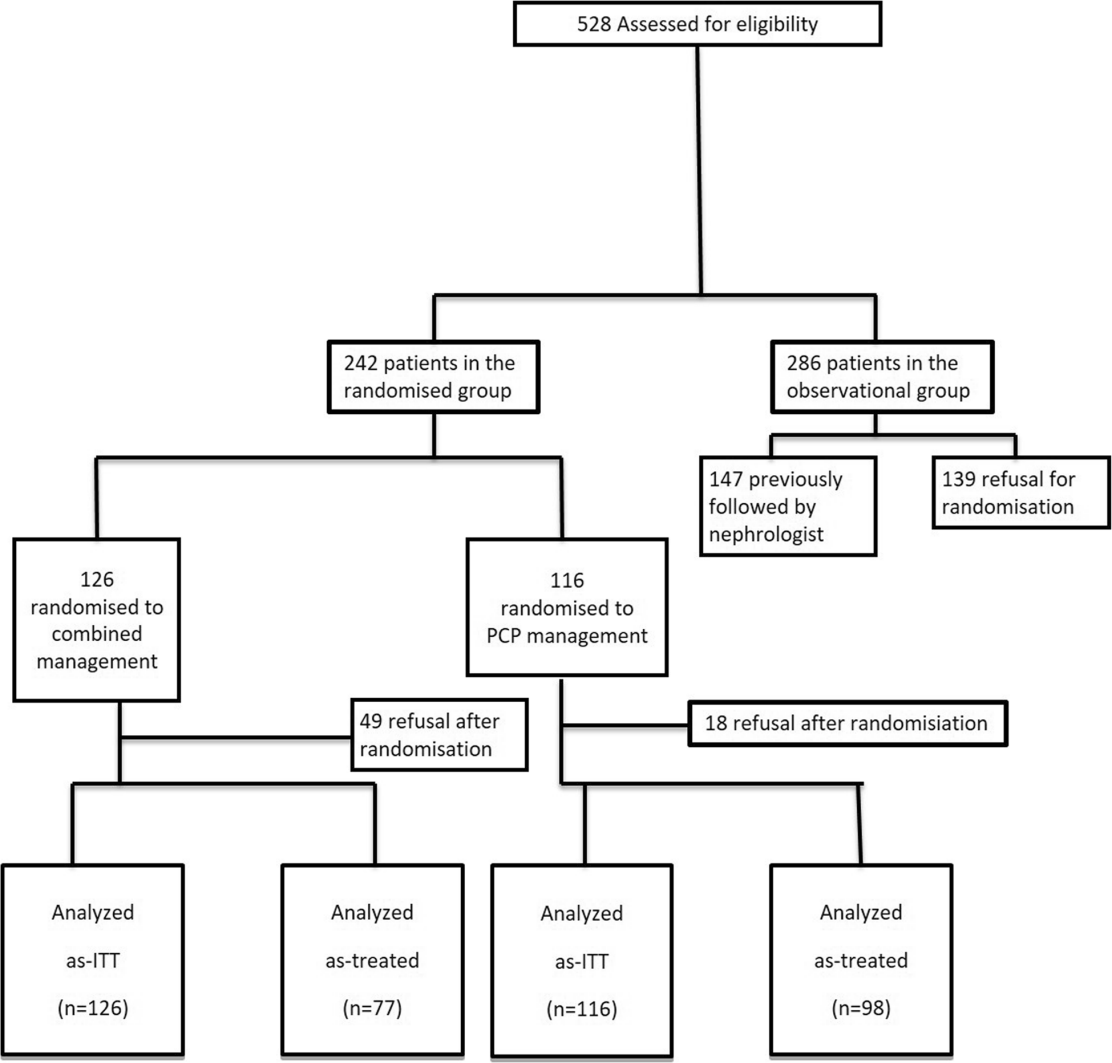
Flow chart profile

We identified 528 patients with an eGFR between 15 and 45 ml/min/1.73m^2^ of whom 139 had refused the randomisation and 147 were already followed by a nephrologist, leaving 242 patients to be randomised (Fig. 1). Within the PCP management group, only 4 patients were followed by the Community Medicine clinic. In this group of the 116 patients followed for a mean period of 51 months, there were < 10 phone calls asking for advice.

After inclusion, 67 patients dropped out from the study. Mean follow-up was 51 + 20 months.

### Baseline demographics

Hospitalized patients with CKD were characterized by advanced age, and high rates of vascular/diabetic nephropathy and elevated comorbidity scores.

Patients who were already followed by a nephrologist (*n* = 147), were younger, had a lower eGFR at baseline and had less vascular/diabetic nephropathy. Patients who refused to be randomized (*n* = 139) were older and women were more prevalent (Table 3) in this group.

**Table 3:** Baseline characteristics of screened CKD patients

Characteristics	Overall (***N*** = 528)	Included (***N*** = 242)	Excluded (***N*** = 286)		p
			Previously followed (N = 147)	Declined participation (N = 139)	
Mean age (years)	70 +/− 9	70 +/− 8	66 +/− 12	74 +/− 5^a^	0.001
Male gender	323 (62%)	169 (70%)^a^	82 (57%)	72 (52%)	0.003
Diabetes/vascular nephropathy	415 (79%)	203 (85%)	86 (59%)^a^	136 (90%)	0.001
Charlson score	4.9 (1.9)	4.9 (2.4)	5.2 (1.5)	4.8 (1.5)	0.06
eGFR (ml/min/1.73m^2^)	32 (9.3)	34 (8)	26 (10)^a^	37 (7)	0.001

Continuous variables are expressed as mean (SD)

Categorical variables are expressed as n (%)

^a^statistically different when compared to the two other groups

### Primary outcome: death or rehospitalisation

**Fig. 2:**
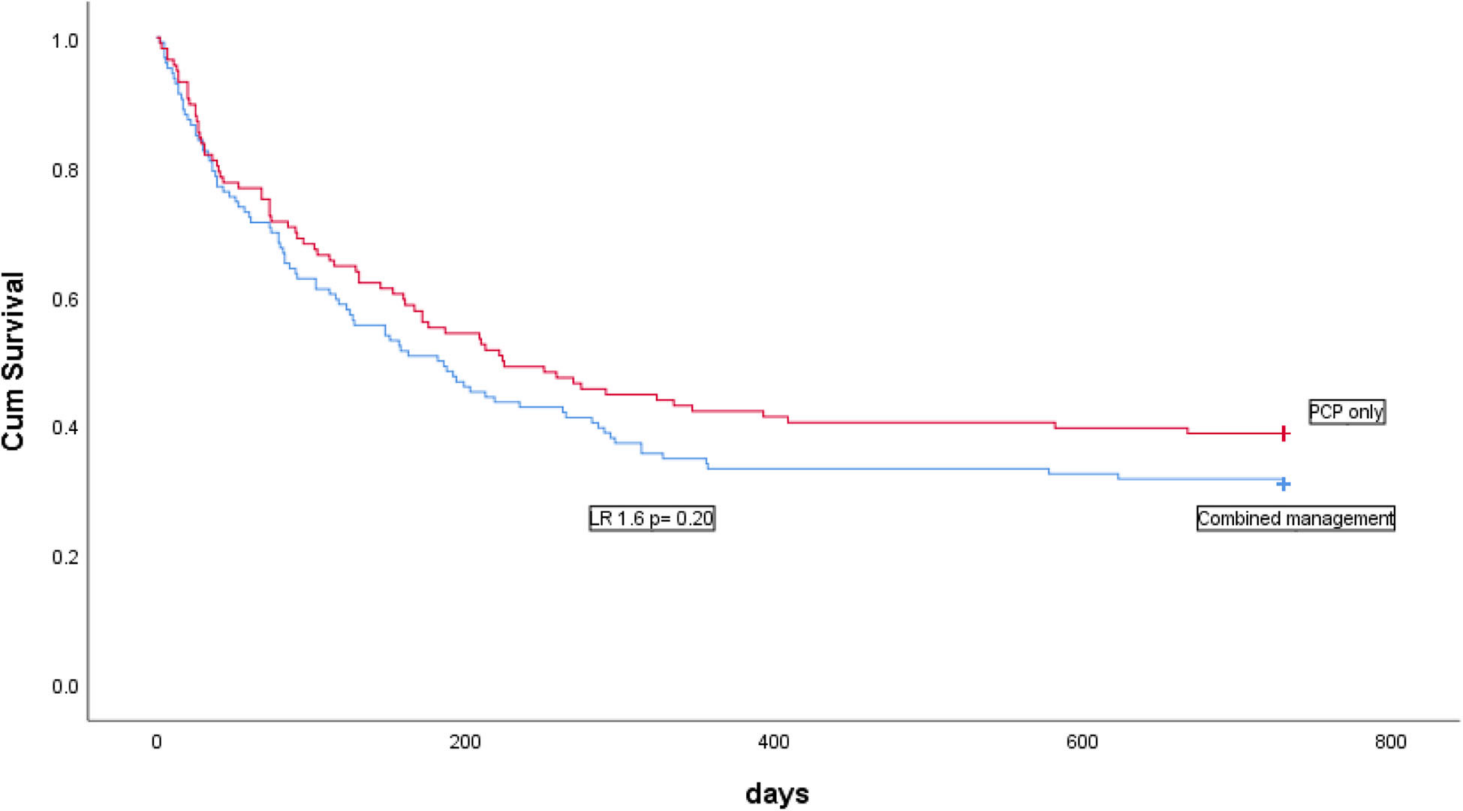
Kaplan-Meier analysis comparing 2-yr survival without hospitalisation. Between patients with PC management versus co-management

The combined primary outcome was attained in 61% (71/116) and 69% (87/126) of the patients in the PCPs and combined management groups (*p* = 0.25). There was no difference in the 2-year survival without rehospitalisation rate between the 2 groups (Fig. 2, log rank test = 0.2). The 2-yr survival was 83 and 84% in the PCPs and combined management groups (*p* = 0.80) respectively, and the incidence of emergency hospitalisation within this time period was respectively 38 and 47% (*p* = 0.15). In multivariate analysis, the comorbidity score was the only variable associated with death and/or rehospitalisation (Table 4).

**Table 4:** Cox regression analysis for composite outcome (death or readmission)

Characteristics	Unadjusted model		Adjusted model	
	HR (95% CI)	p	HR (95% CI)	p
Age	0.99 (0.97–1.00)	0.14	0.98 (0.97–1.000)	0.08
Male gender	1.18 (0.83–1.67)	0.37	1.10 (0.77–1.57)	0.62
Charlson score	1.17 (1.08–1.37)	0.04	1.09 (1.01–1.19	0.04
eGFR	0.99 (0.97–1.00)	0.11	0.99 (0.97–1.01)	0. 18
Combined management ^a^	1.22 (0.90–1.68)	0.20	1.23 (0.89–1.70)	0.21

^a^Reference is PCP management only

### Secondary outcomes

Renal replacement therapy was implemented in 12 and 13 patients in the PCPs and combined management groups, respectively. Rate of emergency RRT versus planned or no dialysis was similar in both groups (9 and 7% in PCPs and combined management groups respectively, *p* = 0.62). Emergency RRT was associated with age, baseline eGFR and comorbidity score (Table 5).

**Table 5:** Cox regression analysis for planned versus emergency RRT

Characteristics	Unadjusted model		Adjusted model	
	HR (95% CI)	p	HR (95% CI)	p
Age	0.94 (0.91–0.98)	0.001	0.92 (0.88–0.96)	0.001
Male gender	4.19 (0.96–18.2)	0.06	2.25 (0.50–10.20)	0.29
Charlson score	1.22 (0.97–1.53)	0.09	1.42 (1.19–1.70)	0.001
eGFR	0.92 (0.88–0.97)	0.002	0.93 (0.88–0.98)	0.005
Combined management ^a^	0.67 (0.27–1.71)	0.40	1.99 (0.72–1.48)	0.18

^a^Reference is PCP management only

### Laboratory and clinical parameters evolution

In Table 6 are shown all the characteristics evolution at baseline (T0) and 1–2 years after randomization (T1-T2). There was no difference in the blood pressure measures, neither clinical nor over 24 h.

**Table 6:** Clinical and laboratory data evolution over two-year follow-up

Characteristics	PCP management only			Combined management			P*
	T0 (***N*** = 116)	T1 (***N*** = 99)	T2 (***N*** = 93)	T0 (***N*** = 126)	T1 (***N*** = 71)	T2 (***N*** = 85)	
**Blood pressure measurement**							
Office SBP (mmHg)	127 (26)	132 (33)	125 (43)	131 (23)	137 (19)	135 (26)	0.65
Office DBP (mmHg)	72 (15)	71 (18)	69 (24)	74 (14)	78 (12)	75 (14)	0.31
ABPM SBP (mmHg)	132 (24)	135 (27)	135 (17)	132 (17)	133 (15)	126 (43)	0.20
ABPM DBP (mmHg)	74 (13)	73 (11)	75 (10)	79 (12)	78 (11)	76 (10)	0.20
**Transthoracic ultrasound**							
LVH^a^	31/97 (32%)	8/29 (28%)	4/29 (14%)	42/112 (38%)	22/62 (35%)	20/50 (40%)	0.22
EF (%)	54 (14)	36 (27)	55 (17)	55 (17)	43 (29)	57 (16)	0.21
**Laboratory data**							
Creatinine (mmol/l)	175 (53)	203 (99)	221 (143)	176 (75)	184 (104)	212 (149)	0.45
eGFR (ml/min)	34 (80)	32 (15)	31 (16)	34 (90)	35 (13)	32 (14)	0.65
Hemoglobin* (g/l)	112 (17)	117 (18)	114 (26)	111 (20)	128 (20)	124 (30)	**< 0.001**
Ferritin (g/l)	102 (60–188)	99 (41–241)	215 (88–494)	176 (81–405)	148 (74–267)	137 (82–234)	0.35
Transferrin sat. (%)	24 (19)	24 (18)	27 (23)	21 (13)	26 (10)	23 (11)	0.44
Calcium (mmol/l)	2.4 (0.2)	2.3 (0.4)	2.3 (0.4)	2.4 (0.2)	2.4 (0.1)	2.3 (0.4)	0.33
Phosphate (mmol/l)	1.1 (0.3)	1.2 (0.2)	1.3 (0.4)	1.2 +/−0.2	1.2 +/− 0.2	1.3 +/− 0.3	0.57
PTH* (pmol/l)	9 (7–13)	11 (8–17)	11 (8–32)	8 (4–14)	10 (6–13)	9 (5–15)	**0.002**
Bicarbonate (mmol/l)	25 (5)	24 (6)	26 (6)	25 (5)	25 (4)	25 (4)	0.56
Albumin (g/l)	29 (5.4)	34 (6)	33 (6)	35 (5)	35 (5)	36 (4)	0.19
24-h proteinuria (g/l)	0.3 (0.2–3.8)	0.5 (0.1–2.0)	1.5 (0.3–5.3)	0.4 (0.2–1.0)	0.3 (0.1–1.2)	0.4 (0.2–1.1)	0.97
**Medications**							
RAS blockers	68 (59%)	36 (84%)	27 (68%)	92 (73%)	54 (82%)	49 (84%)	0.82
Diuretics*	62 (53%)	34 (79%)	31 (78%)	81 (64%)	40 (61%)	40 (69%)	**0.007**
Beta-blockers	70 (56%)	26 (60%)	22 (55%)	68 (59%)	39 (59%)	32 (55%)	0.06
Statin	65 (56%)	25 (58%)	19 (48%)	64 (51%)	40 (61%)	35 (60%)	0.11
Aspirin	2 (2%)	2 (5%)	1 (3%)	5 (4%)	9 (14%)	8 (14%)	0.90
Insulin	36 (31%)	16 (37%)	13 (33%)	32 (25%)	20 (30%)	14 (25%)	0.54
Oral antidiabetic	23 (20%)	10 (23%)	8 (20%)	23 (18%)	14 (21%)	9 (16%)	0.28
Erythropoietin	8 (7%)	8 (12%)	2 (5%)	11 (9%)	7 (11%)	6 (10%)	0.36
Iron	2 (2%)	3 (7%)	1 (3%)	7 (6%)	7 (11%)	6 (10%)	0.77
Vitamin D	19 (16%)	11 (26%)	11 (28%)	13 (10%)	18 (27%)	16 (28%)	0.13

*Significant *p*-value of the interaction between time and random group: significant p-value means a difference in the evolution of the 2 groups

Continuous variables are expressed as mean (SD) or median (IQR) depending on distribution. Categorical variables are expressed as n (%)

T0, T1 and T2 represent baseline and follow-up at one and two years respectively

^a^As some patients did not have follow-up echocardiography, results are expressed as n/n (%)

To note, only 42 patients in the co-management and 18 in the PCP group had undergone the 3 planned 24 h- ABPM.

There was also no difference in left ventricular hypertrophy (LVH) rate or cardiac ejection fraction. However, the number of patients which underwent echocardiography dropped from 209 at baseline to 79 (29 in the PCP only management group) at two years and these results should be interpreted with caution.

No significant change in the laboratory data at two years was observed in levels of: creatinine, eGFR, ferritin, transferrin saturation, calcium, phosphate, bicarbonate, albumin and proteinuria evolution. There were only a statistically significant higher level of hemoglobin in the co-management group *p* < 0.001) and a lower level in PTH as well after randomization (*p* = 0.002).

Regarding the medications, the majority of patients in both groups were on Renin-Angiotensin blockers (RABs), diuretics and beta-blockers at 2 years follow-up. However, patients in the co-management group were less treated by diuretics after randomization (*p* = 0.007). For the rest of the medication, there was no difference between the 2 groups in aspirin, insulin oral antidiabetics, iron and vitamin D prescriptions after randomization.

### Survival between randomised and non-randomised patients

**Fig. 3:**
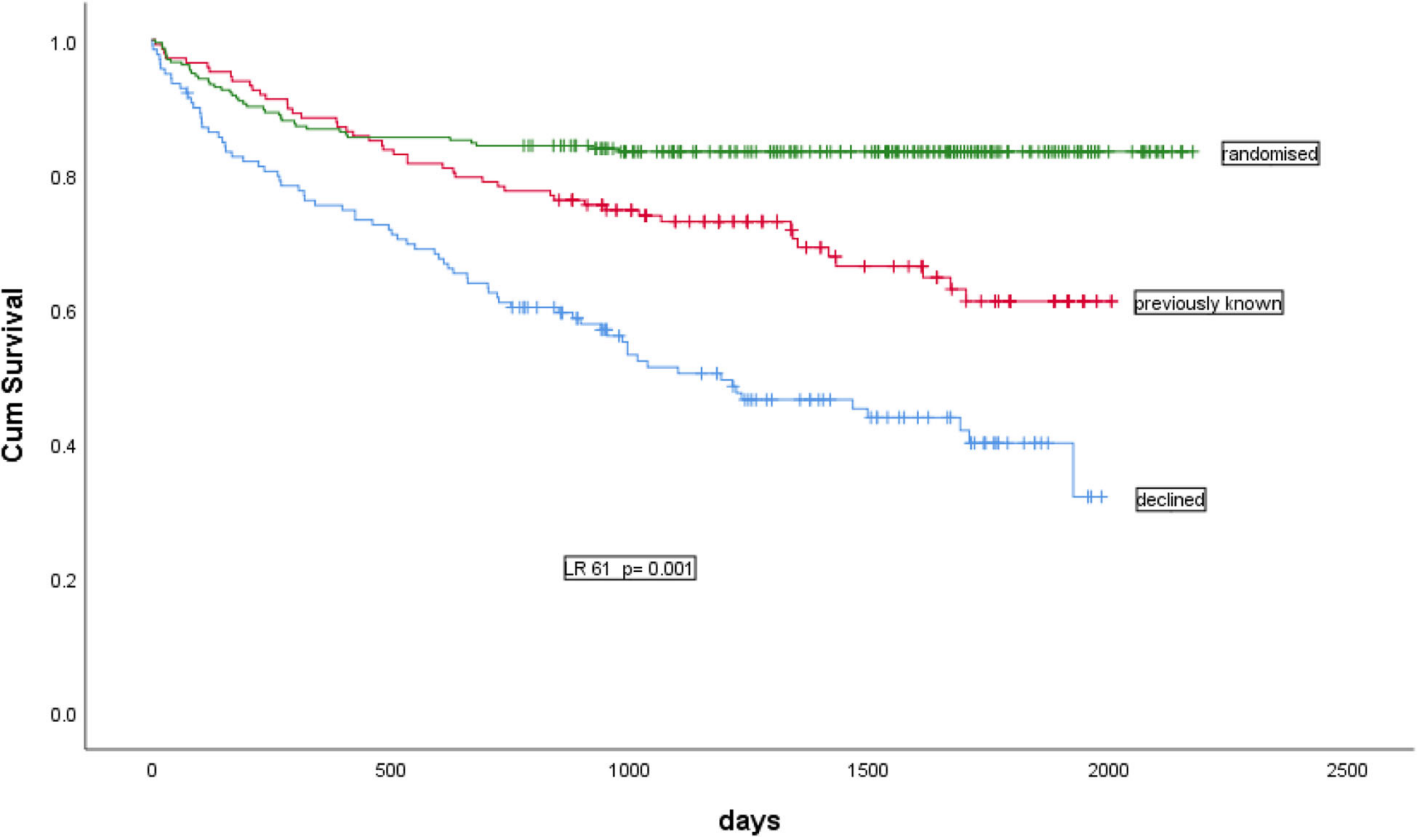
Kaplan-Meier analysis comparing survival between randomized patients, patients with previous nephrologist management and those who declined to participate

Patients who declined the study or were excluded on account of previous follow-up by nephrologists had a statistically significant decreased survival. Long-term mortality was 54% in the refusal group, 32% in those previously known by nephrologists as compared to 16.5% in the randomized group (*p* < 0.001) (Fig. 3).

### KDQOL results

Among 122 patients who answered the questionnaire at baseline and follow-up, the means of all KDQOL scales were similar in the two trial arms at baseline, with few changes as follow-up progressed (Table 7).

**Table 7:** KDQOL scale values among patients who returned a baseline assessment and at least one follow-up assessment

KDQOL scale	Time	Combined management (***N*** = 62)		PCP management only (***N*** = 60)		p
		n	Value	n	Value	
Symptoms	T0	62	70.0 (19.8)	60	70.6 (18.9)	0.87
	T1	61	74.4 (16.7)	57	70.4 (19.0)	0.22
	T2	50	72.7 (17.7)	49	72.2 (20.0)	0.90
Effects of kidney disease	T0	59	79.5 (21.7)	56	76.5 (19.4)	0.45
	T1	59	81.4 (18.4)	51	75.7 (24.4)	0.17
	T2	48	80.0 (18.8)	47	80.9 (19.4)	0.82
Burden of kidney disease	T0	59	70.8 (30.1)	60	63.2 (28.7)	0.16
	T1	60	70.3 (31.1)	57	74.3 (28.3)	0.48
	T2	50	68.2 (29.5)	47	70.2 (30.5)	0.74
Work status	T0	61	31.1 (31.8)	57	28.9 (34.0)	0.72
	T1	58	28.4 (32.6)	53	25.5 (32.0)	0.63
	T2	49	27.6 (33.9)	48	26.0 (32.6)	0.82
Cognitive function	T0	61	79.3 (24.2)	60	75.8 (22.5)	0.41
	T1	60	76.2 (23.3)	56	75.7 (22.0)	0.92
	T2	50	76.8 (23.6)	46	77.0 (23.9)	0.97
Quality of social interaction	T0	62	78.3 (18.1)	60	80.6 (18.2)	0.49
	T1	61	78.0 (17.5)	57	78.1 (18.2)	0.98
	T2	50	78.7 (18.7)	48	79.8 (16.6)	0.75
Sexual function	T0	25	54.5 (39.5)	23	62.5 (36.0)	0.47
	T1	26	62.5 (37.9)	20	68.1 (39.6)	0.63
	T2	18	66.7 (37.4)	13	70.2 (40.0)	0.80

Continuous variables are expressed as mean (SD)

T0, T1 and T2 represent baseline and follow-up at one and two years respectively

## Discussion

In patients with moderate to severe CKD, co-management by PCPs and nephrologists as compared to management by PCPs who received guidelines and had access to advices from nephrologists, did not bring substantial improvement in survival without hospitalization. The very few phone advice phone calls in the standard care group suggest that the nephrologist input was not a mitigating factor in our study.

Cardio-protective medications such as aspirin and statins were prescribed in the co-management group than in the PCP only management group similarly. A majority of patients in both groups used RABs with subsequent good BP control as shown in ABPM results. Improved BP control may be of importance in this group, as it has been shown to improve CV mortality after onset of ESRD [24]. Although BP is often poorly managed and controlled in CKD patients [25], our patients differed from the real practice world as their baseline BP patterns were already within normal range. This may explain why there was no demonstrable LVH regression and evolution of renal functions remained identical in both groups at 2-year follow-up. The co-management did neither translate into an improved nephrological outcome in terms of renal function decline and planned RRT rate nor into a lower mortality and recurrence of hospitalisations at two-years follow-up. A good adhesion of PCPs to the guidelines may have contributed to this absence of differences and that may also explain the similar evolution in most of the laboratory parameters during this study between the two groups. Our results are in agreement with those of the CRIC cohort in which patients previously seen by a nephrologist had a more frequent use of RABs but no positive effect on recommended guideline targets or outcomes [26].

Of interest, patients who either declined to participate in this study or were excluded on account of a previous referral to a nephrologist had a much grimmer prognosis in terms of life expectancy. Baseline characteristics were slightly different in these two groups as there were older and more female patients in the refusal group and mean eGFR was lower in patients already known by nephrologists. Regarding the patients previously followed by a nephrologist, their mortality rate was significantly higher as compared to those randomized. This probably reflects the fact that patients participating in a clinical trial do not correspond to those in the “real practice” world.

Quality of life (QoL) has not been extensively studied in CKD patients and there is a scarcity of longitudinal studies investigating the change of QoL over the time course of CKD [27]. Lately, QoL was found to be associated in CKD patients with shorter and also long durations of sleep, which could impact notoriously CKD progression [28]. Within this 2-year time interval, we did not observe any substantial change in QoL, and no difference between the two treatment groups.

Regarding the expectations of CKD patients in terms of research, it was shown in a survey that most of them favour research on treatments aimed at stopping progression of CKD [29]. Many models of care have therefore been developed during the last 20 years in order to better slow progression to ESRD in CKD patients.

A real emphasis has recently been put on integrated care or nurse-driven care to increase patients’ compliance but the nurse-coordinated or multidisciplinary models gave conflicting results [30–33]. The Masterplan study enrolled 740 CKD patients who were randomized to usual standard care by a nephrologist or reinforced by nurse practitioner care. With 5. 7 years of follow-up, this trial demonstrated a lesser decrease of renal function, although cardiovascular mortality did not change substantially [34, 35]. This study also showed that lifestyle interventions were difficult to maintain over a long period of time due to changing emphasis on goals and less bonding of nurses with patients [36]. Strategies encouraging integrated care implicating healthcare officers seems to be more effective in developing countries with measurable delay of CKD [37].

Although a simplified model of collaborative care between GPs and nephrologists is favoured by both professions, the quest for a model which combines efficiency and human resources cost-sparing remains to be defined [38] .

Our trial had several limitations. First, we did not enroll the number of patients which was determined in our sample size calculation within the time period allowed for recruitment and follow-up time was limited to two years. So, a lack of power (type 2) error may be possible although our results are in agreement with previous studies. We had a 27% drop-out rate after randomization, especially in the co-management group which highlights the difficulty of comparing two managements, since one implied more medical appointments. Per protocol analysis of our results was however similar (data not shown) to our intent-to-treat analysis. We could only get a 24 h-ABPM in a small subset of patients followed only by PCPs and our statement about similar BP control in the two groups should be accepted with caution. Although we recommend dietary advice in our guidelines, no real emphasis was put on low-protein diets supplemented with ketoanalogues or very low-sodium diet and these interventions have been proven powerful on the short-term to delay progression to ESRD [39, 40].

## Conclusions

Though our results did not show an advantage of a scheduled nephrologist care in this frail population such as the CKD patients, these data should be put in perspective of our study’s limitations but also hint that PCPs made aware of guidelines could obtain similar results as renal physicians in the care of CKD patients. In our study, patients considered at moderate risk of rapid renal progression were deemed stable enough to be safely managed by a general practitioner receiving written instructions and consultations by nephrologists on demand. By contrast, the increased mortality rate observed in patients who did not participate in the trial underlines the need of a regular medical follow-up and the benefit of a close collaboration between PCPs and nephrologists. With the perspective of an augmentation of CKD patients, these results are reassuring and should encourage the nephrologists to develop network with PCPs on regional base to implement guidelines and strategies of follow-up of CKD patients. The discrepancy in the mortality rate observed in patients randomized and those who did not participate in the trial also underlines the chasm separating the worlds of RCTs and of real practice. Our results should encourage more studies including all professionals involved in the care of CKD patients in order to improve the interdisciplinary collaboration and our patients’ prognosis.

## Supplementary information

**Additional file 1.** SPPS file (data from anonymized patients randomized in the study).

**Additional file 2.** SPPS file (data from anonymized patients in the observational study).

## Data Availability

SPSS anonymised datasets are provided in supplementary files.

## Abbreviations

ABPM: Ambulatory blood pressure monitoring
ACEIs: Angiotensin-converting enzyme inhibitors
ACEIs: Angiotensin-converting enzyme inhibitors
AKI: Acute kidney injury
ARBs: Angiotensin-receptor blockers
ARBs: Angiotensin-receptor blockers
BNP: Brain natriuretic peptide
BP: Blood pressure
CCl: Creatinine clearance
CKD: Chronic kidney disease
CRP: C-reactive protein
CVD: Cardiovascular disease
DBP: Diastolic blood pressure
EBPG: European Best Practice Guidelines
EF: Ejection fraction
ESRD: End stage renal disease
GFR: Glomerular filtration rate
Hb: Hemoglobin
HD: Haemodialysis
KDIGO: Kidney Disease Improving Global outcomes
KDQOL: Kidney Disease Quality of Life
LDL: LDL, low density lipoprotein
LVH: Left ventricular hypertrophy
NSAIDs: Non-steroidal anti-inflammatory drugs
PCPs: Primary care physicians
PD: Peritoneal dialysis
PTH: Parathyroid hormone
RAS: Renin-angiotensin system
RRT: Renal replacement therapy
SBP: Systolic blood pressure
TX: Renal transplantation
